# Mechanisms of Action of Exclusive Enteral Nutrition and Other Nutritional Therapies in Crohn’s Disease

**DOI:** 10.3390/nu16213581

**Published:** 2024-10-22

**Authors:** Ramasatyaveni Geesala, Pratik Gongloor, Neeraja Recharla, Xuan-Zheng Shi

**Affiliations:** 1 Department of Internal Medicine, The University of Texas Medical Branch, Galveston, TX 77555, USA; rageesal@utmb.edu (R.G.); nerechar@utmb.edu (N.R.); 2 John Sealy School of Medicine, The University of Texas Medical Branch, Galveston, TX 77555, USA; prgonglo@utmb.edu

**Keywords:** inflammatory bowel disease, Crohn’s disease, nutritional therapy, exclusive enteral nutrition, Crohn’s disease exclusive diet, specific carbohydrate diet, FODMAP diet, semi-vegetarian diet, mechanical stress, microbiota

## Abstract

Background and Objectives: Crohn’s disease (CD) is an inflammatory bowel disease (IBD) characterized by transmural inflammation and intestinal fibrosis involving mostly the small intestine and colon. The pathogenic mechanisms of CD remain incompletely understood and cures are unavailable. Current medical therapies are aimed at inducing prolonged remission. Most of the medical therapies such as corticosteroids have substantial adverse effects. Consequently, many dietary therapies have been explored for the management of CD. Up to now, exclusive enteral nutrition (EEN) has been considered the only established dietary treatment for IBD, especially CD. In this article, we aim to give a concise review about the current therapeutic options and challenges in the management of CD and aim to compare the efficacy of EEN with other dietary therapies and update on the possible mechanisms of the benefits of EEN and other nutritional therapies. Methods: We searched the literature up to August 2024 through PubMed, Web of Science, and other sources using search terms such as EEN, nutritional therapy, IBD, Crohn’s disease, ulcerative colitis. Clinical studies in patients and preclinical studies in rodent models of IBD were included in the summary of the therapeutic benefits. Results and Conclusions: EEN involves oral or nasogastric tube feeding of a complete liquid diet with exclusion of normal foods for a defined period (usually 6 to 8 weeks). EEN treatment is demonstrated to have anti-inflammatory and healing effects in CD through various potential pathways, including altering gut bacteria and their metabolites, restoring the barrier function, direct anti-inflammatory action, and indirect anti-inflammatory action by eliminating mechanical stress in the bowel. However, efficacy of other nutritional therapies is not well established in CD, and mechanisms of action are largely unknown.

## 1. Current Understanding of Crohn’s Disease Etiology and Pathophysiology

Inflammatory bowel disease (IBD) is a chronic inflammatory disease of the gastrointestinal (GI) tract. The two main types of IBD are Crohn’s disease (CD) and ulcerative colitis (UC). In the United States, 1.3% of the total population, or nearly 3 million individuals, are diagnosed with IBD [1]. Among the two types of IBDs, CD can affect any part of the GI tract (with the ileum and colon mostly involved) and can lead to transmural inflammation and complications such as strictures or fistulas. UC, on the other hand, primarily affects the colon. CD has a higher incidence rate of 3 to 20 cases per 100,000 per year. The incidence of CD, particularly in children, is on the rise. Although it can occur at any age, up to 25% of CD patients are diagnosed before the age of 20 [1]. While the cause of CD is not fully understood, it is believed to be due to a complex interaction of genetic predisposition, environmental factors, gut microbiome imbalances, and problems with the immune system and gut barrier function.

Genetic factors play a role in CD, with up to 12% of patients having a family history of the disease. Certain populations, such as Ashkenazi Jews, have a higher risk of CD indicating the genetic hereditability of the disease [2]. Genome-wide association studies (GWAS) analyze genetic variations across the entire genome to identify differences between individuals with and without a disease. Early GWAS on CD revealed significant associations with genes involved in autophagy (ATG16L1, IRGM) and immune system pathways (TLR4, CARD9, IL23R, STAT3, HLA, TNFSF15, IRF5, PTPN22) [3]. These studies also highlighted a genetic overlap between CD and other immune-mediated diseases such as ulcerative colitis, type 1 diabetes, celiac disease, and rheumatoid arthritis [3,4].

Approximately 200 alleles have been identified to be linked with IBD, of which 37 were CD-specific. Some of these CD-linked genes, such as NOD2, ATG16L1, LRRK2, IRGM, Il23R, HLA, STAT3, and JAK2, are known to be associated with host-microbe interactions and T helper 17 (Th17) immune signaling pathways [3,5]. However, genetic variations were shown to be causative only in a minority of cases, implying that other factors such as epigenetic mechanisms may contribute to CD. Environmental factors such as Western lifestyle and diet (high in saturated fat, low in fiber), childhood antibiotic use, and smoking have been linked to CD [6].

Gut microbiome, the community of microorganisms in the GI tract, is crucial for the maintenance of gut homeostasis. Several reports have shown differences in the types and amounts of bacteria in the gut of CD patients compared to healthy individuals. Disruption of gut microbiome, or dysbiosis, is a common characteristic of CD pathogenesis. It is evident by reduction in commensal species like Bacteroides, Firmicutes, and *Faecalibacterium prausnitzii* species in CD patients. However, other microbial species such as Gamma proteobacteria and Actinobacteria were found increased in CD patients. About one-third of CD patients have shown increased abundance of pathogenic *Escherichia coli* [7,8,9]. *Enterobacteriaceae* is an adherent-invasive strain and has the ability to cross the mucosal barrier and replicate within macrophages to induce secretion of tumor necrosis factor-α (TNF-α) [10]. Although microbiota manipulation is a growing research field, the evidence thus far is still too limited to propose probiotics and prebiotics as a treatment option for CD.

Defects in the gut barrier, which normally prevents harmful substances from entering the body, can also contribute to CD. Emulsifiers in processed foods, along with genetic mutations and problems with autophagy (a cellular cleaning process), can damage the gut barrier and trigger inflammation [6]. It is still unclear whether a compromised mucosal barrier is a cause or consequence of inflammation in CD. However, research suggests that increased intestinal permeability may be an early factor in CD development. This is supported by studies showing heightened permeability in patients with inactive CD, a uniform increase in permeability in minimally damaged colon tissue from CD patients [10], and the detection of increased permeability before inflammation in animal models of CD [11].

The immune system is dysregulated in CD. Innate lymphoid cells (ILCs), which help maintain the gut barrier, are involved in the disease process. When harmful substances enter the gut, immune cells produce inflammatory molecules like TNF-alpha, interleukin-17 (IL-17), IL-22, and interferon-gamma. Specific types of ILCs, i.e., ILC1 and ILC3, are particularly relevant to CD [12,13].

Th17 cells are also implicated in CD. Traditionally, CD has been associated with a Th1 cytokine profile, while Th2 cytokines are modulators of ulcerative colitis. This concept has been challenged by the description of tolerizing regulatory T cells (Treg) and by proinflammatory Th17 cells, a novel T cell population characterized by the master transcription factor RORgt, the surface markers IL23R and CCR6, and by the production of proinflammatory cytokines IL17A, IL17F, IL21, IL22, and IL26, and the chemokine CCL20. Th17 cells differentiate under the influence of IL1β, IL6, IL21, and IL23. Previous studies have revealed that there is a positive correlation between IBD severity and IL-17 levels secreted by PBMCs in IBD patients [14]. Several studies have demonstrated an important role of Th17 cells in intestinal inflammation, particularly in CD [5,12,13,14]. Furthermore, IBD genome-wide association studies have shown several polymorphisms in Th17-related genes [5].

## 2. Treatment Options and Challenges in the Management of Crohn’s Disease

CD can manifest in different ways, including inflammation, stricture formation, and fistulas. It often affects the terminal ileum and colon, but less frequently involves the rest of the GI tract, even the mouth. Symptoms can range from abdominal pain and diarrhea to extra-intestinal manifestations like eye problems, blood disorders, joint pain, and skin issues [15].

CD is marked by an excessive influx of leukocytes into the inflamed intestinal segment(s) and a high level of secreted proinflammatory cytokines. Current medication regimens aim to reduce inflammation by using immunomodulators or immunosuppressants such as corticosteroids, methotrexates, thiopurines, and biologics (i.e., anti-TNF) to suppress immune system activity. Currently, the FDA has approved four anti-TNF agents (infliximab, adalimumab, golimumab, and certolizumab pegol). Other biologic agents targeted to treat CD are monoclonal antibodies to certain integrins (α4 or α4b7) or interleukins (IL-12/IL-23). Depending on the disease severity, treatment options range from 5-aminosalicylic acid and corticosteroids to biologics and immunomodulators [16,17]. However, these medications can cause various side effects, including an increased risk of infections and malignancy. In children with CD, the use of corticosteroids is particularly concerning due to their association with growth retardation and reduced bone accrual [18]. In many cases with severe inflammation, stricture formation, or fistulas, surgery may be necessary. But surgery is not a cure, as recurrence may occur even after surgical treatments.

Extensive research has been conducted to understand the connection between changes in the gut microbiome and active CD or response to treatment. Manipulating the microbiome with antibiotics, such as azithromycin and metronidazole, has shown potential in treating pediatric CD [19,20]. Antibiotics are also used to help maintain remission in perianal CD when combined with anti-TNF therapy [21]. However, despite the benefits of immunosuppressants and antibiotics, a significant number of patients experience primary nonresponse or loss of response to these treatments, highlighting the need for new and effective therapies for CD.

Recent research suggests that environmental factors, particularly diet, may play a significant role in IBD, especially CD. This has led to dietary interventions being explored as a treatment option. Elemental liquid diets, which are made up of single amino acids, were initially used for preoperative nutritional support in CD. However, the potential therapeutic benefits of the enteral nutrition were noticed when CD patients who took the liquid diet while waiting for surgery showed a reduction of inflammation and improvements in their symptoms and nutritional status [22,23]. Follow-up studies found that enteral nutrition with a non-elementary liquid diet achieved similar benefits as using an elementary diet [22,23,24]. Since then, extensive research has supported the use of nutritional therapy in CD, although the exact mechanism of action and ideal formulation remain unclear. Recent European guidelines highlight the importance of dietary therapy, especially exclusive enteral nutrition (EEN), in managing mild-to-moderate CD, while also emphasizing the need for further research on new dietary strategies. EEN, a liquid diet that provides all necessary nutrients, is considered a first-line treatment for pediatric CD [22,23,24]. In addition, other nutritional therapies have been developed and tested in recent years to minimize exposure to foods that may negatively affect the microbiome, intestinal barrier, and innate immunity. In this review, we discuss efficacy, safety, and clinical outcomes of EEN and other nutritional therapies. More importantly, we offer the most recent discoveries about the potential mechanisms of different nutritional therapies as compared to EEN in inducing and maintaining remission in CD.

## 3. Nutritional Therapies in Crohn’s Disease

Diets significantly influence the composition and balance of gut microbiota, which plays a crucial role in metabolism and immune response. Changes in dietary intake, particularly the Western diet high in fats and carbohydrates, have been linked to gut microbiome imbalances (dysbiosis) and an increase in pediatric CD cases. This connection has led to investigations into nutritional therapies as potential treatments for CD. While current medical treatments primarily target inflammation, nutritional therapies aim to correct dysbiosis, restore metabolic balance, and reduce inflammation [16,25].

### 3.1. Enteral Nutrition

Enteral nutrition (EN) involves consuming a complete liquid diet that provides all the necessary calories without including solid food. EN can be administered orally as a drink, or through a feeding tube, with similar effectiveness [26,27]. EN liquid diets for CD can be classified based on the nitrogen source, which can be derived from amino acids or protein. Elemental EN diets are antigen-free, while oligopeptide or semi-elemental EN diets are made by breaking down proteins into shorter chains of amino acids. Polymeric EN diets contain whole proteins from sources like milk, meat, egg, or soy [26,27,28]. Interestingly, EN treatments with either elementary or semi-elementary or polymeric liquid diets all achieve anti-inflammatory effects comparable to corticosteroids in pediatric CD patients [26,27,28]. EN treatment is particularly recommended during flare-ups of Crohn’s disease in pediatric populations, where it is typically used for 6-8 weeks to induce remission. EN has also shown benefits as a maintenance diet during remission phases of CD, when it is combined with a regular diet [29]. This type of treatment, known as maintenance enteral nutrition (MEN), can enhance the positive effects of biological therapies and prevent disease relapse after surgery [30,31,32,33]. Additionally, when used as the sole source of nutrition (exclusive enteral nutrition or EEN), it has been shown to improve nutritional status and bone health in children in addition to its known effect in reducing inflammation [30,34,35,36].

EEN is the primary treatment for mild-to-moderate CD in children and adolescents, leading to remission in 80-85% of cases [37,38,39,40]. It is preferred over steroids due to its ability to promote remission while avoiding the growth-stunting effects of steroids. However, similar success has not been replicated in adults (possibly due to practicalities of use, compliance, and tolerability), where corticosteroids still demonstrate better remission rates compared to EEN [41]. EEN is also suggested for managing complicated CD, such as inflammatory strictures or fistulas [42,43]. Enteral nutrition has also been shown to promote mucosal healing, which is important for long-term remission.

Current consensus from several organizations in the world (i.e., European Crohn’s and Colitis organization, the European Society for Pediatric Gastroenterology Hepatology and Nutrition, and North America Society for Pediatric Gastroenterology Hepatology and Nutrition) recommends clinicians to utilize EEN as a first-line therapy to induce remission in pediatric CD patients wherever possible. It is suggested that corticosteroids, or early immunosuppressive therapy, should be reserved for the patients where EEN is not an option [44,45]. EEN is not recommended for UC patients. Current data does not support a therapeutic role of EEN in the treatment of UC, an IBD with no transmural inflammation or stricture formation as in CD [45].

### 3.2. Parenteral Nutrition

Parenteral nutrition (PN), specifically its exclusive form total parenteral nutrition (TPN), delivers nutrients through a central venous catheter [46,47]. PN, unlike EEN, is systematically given as an alternative to EN to IBD patients with complications. The European Crohn’s and Colitis Organisation (ECCO) recommends PN in CD patients to optimize nutritional status before surgery, as a supplement to EN, or as an alternative when EN is not feasible or contraindicated [48]. PN is often used for malnourished patients with acute inflammation to achieve bowel rest, as well as for managing postoperative complications that hinder gastrointestinal function [49]. It is also employed in cases of bowel obstruction, high-output fistulae, bowel ischemia, severe hemorrhage, anastomotic leak, or active disease-causing gut dysfunction [48]. Research has shown that PN improves various markers of nutritional status and reduces inflammation in CD patients, although it may not always lead to a decrease in disease activity [49,50,51,52]. Despite these benefits and the use of additional therapies, relapses of malabsorption remain common in CD patients who use PN treatment.

### 3.3. Crohn’s Disease Exclusion Diet

Crohn’s Disease Exclusion Diet (CDED) is a whole-food diet combined with partial enteral nutrition (PEN) using a specific formula (MODULENTM IBD, Nestlé) [53]. This structured diet aims to limit exposure to dietary components that may harm the gut microbiome, intestinal barrier, and immune function. Foods like animal fat, certain meats, gluten, maltodextrin, emulsifiers, sulfites, and certain monosaccharides are restricted.

In a study by Sigall-Boneh et al., adults and children with CD following CDED with additional PEN for 50% of their energy needs for 6 weeks, followed by a step-down diet with 25% PEN, achieved high rates of clinical response and remission (around 80%) [53,54]. Notably, most patients who opted out of PEN and only followed CDED also achieved remission, suggesting that PEN may not be essential for remission. A recent randomized controlled trial in adults confirmed this, finding no significant difference in remission rates between CDED with and without PEN [55]. However, PEN is still often included due to its nutritional benefits and as a major source of calcium during CDED therapy [56].

Previous studies in both children and adults with CD have shown that combining CDED with immunomodulator medications can often successfully re-induce remission, even in patients who have stopped responding to biologic therapies [53,57]. This is particularly promising for this group of patients, who urgently need alternative treatment options.

CDED has demonstrated potential as a therapeutic approach for pediatric CD in various scenarios, including inducing remission, maintaining remission, reducing medication use, and as a rescue therapy for those who have lost response to other treatments [54]. However, adhering to the CDED treatment requires significant commitment from patients and parents, as it involves careful planning and preparation of meals according to specific dietary guidelines.

### 3.4. Specific Carbohydrate Diet

The Specific Carbohydrate Diet (SCD), originally developed in the 1920s for celiac disease and later adapted for IBD by Dr. Sidney Haas in 1951 [58], allows the consumption of monosaccharides while excluding disaccharides and most polysaccharides. This diet allows for a variety of foods including meat, eggs, oil, amylose-rich vegetables, low-lactose dairy products (e.g., dry curd cottage cheese, homemade 24-h fermented yogurt), nuts, and fruits. However, it restricts foods like sucrose, maltose, isomaltose, lactose, potatoes, okra, corn, fluid milk, soy, high-lactose cheeses, food additives, and preservatives.

Studies have shown that SCD can improve symptoms and quality of life in patients with Crohn’s disease, and in some cases, maintain remission without the need for medication [59]. In children, SCD has also been shown to promote mucosal healing and normalize inflammatory markers [60,61,62,63]. An ongoing research project, funded by the Patient-Centered Outcomes Research Institute (PCORI), aims to compare the effectiveness of SCD with the Mediterranean diet in achieving symptom remission in CD [64]. The results of this study will provide valuable insights into the potential role of the Mediterranean diet in managing CD.

### 3.5. FODMAP Diet

The low FODMAP diet, which stands for fermentable oligosaccharides, disaccharides, monosaccharides, and polyols, was initially designed for individuals with irritable bowel syndrome (IBS) and later proposed for IBD. This diet restricts short-chain carbohydrates that are poorly absorbed and easily fermented by gut bacteria, often leading to diarrhea, bloating, distension, and abdominal pain [65]. Foods high in fructose (certain fruits like apples, dates, and watermelon), fructans (onions, garlic), and galactans (beans, lentils, legumes) are limited, while sucrose is allowed.

While a low FODMAP diet has been shown to improve gastrointestinal symptoms in some patients [66], there is no evidence suggesting it reduces inflammation or calprotectin levels [67]. The low FODMAP diet is generally recommended for patients with inactive IBD who experience IBS-like symptoms, which can affect up to 57% of those with Crohn’s disease [68]. However, a potential drawback of this diet is the reduced intake of prebiotics such as inulin, fructooligosaccharides, and fructose, which can negatively impact the gut microbiome by reducing the population of beneficial Bifidobacterium [69,70].

### 3.6. Semi-Vegetarian Diet

The semi-vegetarian diet (SVD), also known as “flexitarian”, is a primarily plant-based diet that limits, but does not eliminate, meat and fish. It emphasizes vegetables, fruits, cereals, eggs, yogurt, and milk, while excluding processed and refined foods [71]. A study by Chiba et al. found that CD patients in remission who followed an SVD treatment for 2 years maintained remission at a rate of 94%, compared to 33% of those on a regular diet [72]. This suggests that SVD is effective in preventing relapse.

In a case study by Sandefur et al., a CD patient experienced complete symptom resolution after 40 days on a vegetarian diet and avoidance of processed foods [73]. This patient continued the diet and reported complete mucosal healing after 6 months, with only occasional relapses associated with periods of non-compliance.

Another study by the same group investigated a lacto-ovo-vegetarian diet with additional servings of fish and meat [74]. This diet was particularly high in fiber, exceeding the recommended daily intake for the Japanese population. After 2 years, 92% of patients on this diet maintained remission without the need for biologic drugs, suggesting that a high-fiber diet may be beneficial for some CD patients.

## 4. Mechanisms of Action of EEN and Other Nutritional Therapies for Crohn’s Disease

Nutritional therapies, especially EEN, have demonstrated a remarkable capacity to reduce intestinal inflammation and promote mucosal healing in children with Crohn’s disease [75,76]. While these therapies have proven benefits, the specific ways in which they achieve and maintain remission are still not fully understood [77]. Recent studies suggest several potential mechanisms may account for the beneficial effects of nutritional therapies include improving nutritional status, stimulating anti-inflammatory responses, increasing the production of protective proteins, limiting exposure to harmful antigens, enhancing gut barrier function, attenuating mechanical stress, and modulating the gut microbiome [78,79] (Table 1).

### 4.1. Effect on Intestinal Barrier

The intestinal inflammation seen in active Crohn’s disease often leads to a compromised gut barrier, characterized by increased permeability, a damaged mucus layer, and reduced protection against harmful substances [98]. There is growing evidence that certain dietary components can worsen this barrier dysfunction in CD patients [75,99]. However, nutritional therapies have been shown to improve mucus integrity and reduce permeability in children with CD [22,100,101]. One possible explanation for this effect is that these therapies exclude foods that may negatively impact barrier function, allowing the gut lining to heal [101]. Laboratory studies have shown that adding an EEN-polymeric formula to damaged intestinal cells can restore barrier integrity by preventing the disruption of important proteins involved in cell-to-cell connections [102,103]. Specific nutrients like glutamine, often included in nutritional therapy, have also been linked to improved barrier function [95]. Additionally, nutritional therapies, particularly EEN, by reducing inflammation, can indirectly protect the gut lining from further damage caused by inflammatory molecules [104] (Table 1).

### 4.2. Effect on Microbiota

The gut microbiome plays a crucial role in maintaining overall health, and disruptions in this delicate balance, known as dysbiosis, have been linked to the development and progression of CD [105,106]. Nutritional therapies have shown promise in restoring a healthy gut microbiome in CD patients [25,87]. Leach et al. found that EEN leads to a decrease in bacterial diversity, particularly in the Bacteroides group, and this change was associated with reduced inflammation [107]. With the advent of advanced sequencing technologies, i.e., 16S rRNA sequencing and whole-genome sequencing, our understanding of the relationship between diet and gut microbiota has expanded considerably [90,91]. In a study of pediatric CD patients, 16S rRNA sequencing revealed significant shifts in fecal bacterial communities after just 2 weeks of EEN, including a reduction in Gram-negative bacteria (like Bacteroidetes) and an increase in Gram-positive bacteria (like Firmicutes) [91]. These findings suggest that nutritional therapies with EEN may be able to counteract the dysbiosis characteristic of CD, which is often marked by a decrease in Firmicutes, an increase in Proteobacteria, and a reduction in overall bacterial diversity [108,109].

Changes in gut bacteria composition caused by nutritional therapies like EEN (exclusive enteral nutrition) or CDED+PEN (Crohn’s Disease Exclusion Diet + Partial Enteral Nutrition) have been associated with remission and long-term remission in children with Crohn’s disease [54,110]. A study showed that both diets were effective in inducing remission after 6 weeks, with similar changes in gut bacteria composition: a decrease in Actinobacteria and Proteobacteria and an increase in Clostridia [75]. However, only CDED+PEN was able to maintain remission until week 12, which was linked to sustained changes in gut bacteria, while EEN was not effective in maintaining remission and saw a rebound in Proteobacteria [75].

Interestingly, EEN therapy was initially reported to decrease gut bacteria diversity [88,89] in pediatric CD patients. This diversity returns after stopping EEN [88,111]. However, diversity alone may not be the best indicator of gut health, as patients who do not respond to therapy also show changes in gut bacteria composition, but with an increase in Proteobacteria and a decrease in overall species richness [112]. Importantly, a few recent studies, including our own group, found [44] that EEN treatment increased microbial diversity and richness in preclinical studies. In a tightly controlled pre-clinical study, we found that EEN administration significantly improved body weight, inflammation scores, and disease activity index in rats with CD-like colitis induced by intracolonic instillation of TNBS [44]. The mRNA expression of IL-17A and interferon-γ was significantly increased in the colonic tissue in CD rats when fed with regular food. However, EEN treatment significantly attenuated the increase in IL-17A and interferon-γ. Our 16S rRNA sequencing analysis found that gut microbiota diversity and compositions were significantly altered in CD rats, compared to controls. However, EEN treatment improved alpha diversity and increased certain beneficial bacteria such as *Lactobacillus* and *Dubosiella* and decreased bacteria such as *Bacteroides* and *Enterorhabdus*, compared to CD rats with the regular pellet diet. These effects may contribute to the reduced inflammation by EEN in the rat model of CD-like colitis (Table 1).

Bile acids (BAs), which play a key role in regulating the immune response and gut bacteria composition, are also affected in CD. Patients with CD often have altered BA metabolism, with higher levels of primary BAs and lower levels of secondary BAs compared to healthy individuals [113,114]. Research has shown that EEN-induced remission is associated with changes in fecal BAs, with higher levels of primary BAs in patients who do not respond to EEN or not maintain remission [115]. Patients who successfully achieved and maintained remission with EEN showed a predominance of secondary BAs. This finding is notable as the gut bacteria composition differed significantly between those with predominantly primary BAs versus those with secondary BAs [115]. While primary BA-dominant samples mainly contained bacteria like *Bacteroides plebeius*, *Enterobacteriaceae*, *Roseburia*, *Ruminococcus gnavus*, and *Megamonas*, secondary BA-dominant samples were characterized by different Bacteroides species (including *B. uniformis*), *Ruminococcaceae*, *Erysipelotrichaceae*, *Rikenellaceae*, and *Lachnospiraceae* [115].

Gut microbiota is involved in many metabolic pathways of nutrients. Amino acid tryptophan is metabolized in the intestine, leading to kynurenine, serotonin, and indole derivative synthesis under the direct or indirect involvement of the microbiota [116]. Recent studies found that reduction in specific kynurenine pathway compounds and the increase in serotonin pathway compounds are associated with EN-induced and sustained remission in CD [117]. Further research is needed to explore the impact of nutritional therapies on various metabolic pathways implicated in CD, such as tryptophan, and sphingolipid metabolism, and to determine the role of gut microbiota in the impact [116,117,118].

### 4.3. Effect on Inflammation

Nutritional therapies such as EEN and CDED have been shown to effectively reduce intestinal inflammation in children with Crohn’s disease, as evidenced by decreases in inflammatory markers like CRP and calprotectin [75]. This anti-inflammatory effect has been observed in several studies [76]. Research by Logan et al., Breese et al., and Beattie et al. demonstrated that EEN lowers the number of cytokine-producing cells in the intestinal lining of CD patients [119,120]. Furthermore, both polymeric and elemental EEN diets have been shown to decrease gene and protein expression levels of pro-inflammatory cytokines like IL-1β, IFN-γ, and TNF-α in CD patients [121,122]. Additionally, EEN enhances the ability of immune cells from children with CD to respond to anti-inflammatory signals, further supporting its role in reducing inflammation [91]. EEN appears to enhance the activity of TGF-β, a cytokine that suppresses inflammation and promotes the development of regulatory T cells (Tregs), which help to dampen the immune response [123,124,125,126]. Research has shown that EEN increases the number of Tregs and reduces pro-inflammatory Th1 cells in children with CD, suggesting a potential mechanism for its anti-inflammatory effects [91].

Changes in microRNAs, molecules that regulate gene expression, have also been proposed as a factor in the anti-inflammatory effects of nutritional therapies in CD patients [127]. Studies have shown that EEN therapy can normalize levels of these microRNAs in the intestinal lining, suggesting a potential role in gene regulation [128]. While EEN is low in fiber and may reduce levels of beneficial short-chain fatty acids like butyrate, it has been shown to increase the production of proteins like intestinal alkaline phosphatase (IAP) and CEACAM-6, which play a crucial role in the gut’s immune defense against pathogens [97,129,130] (Table 1). While promising, further investigation is needed to fully understand these complex processes.

### 4.4. Elimination of Mechanical Stress

**Figure 1:**
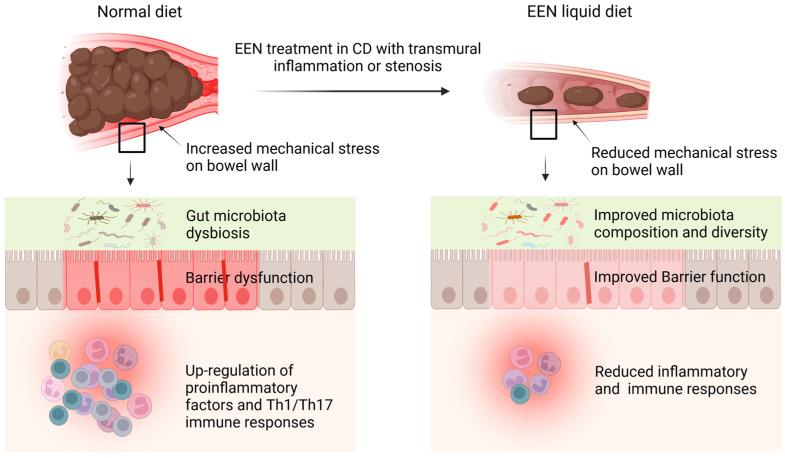
Proposed mechanisms of action of EEN in Crohn’s disease (CD). Transmural inflammation and stenosis in CD lead to increased mechanical stress (MS) in the inflamed tissue and the segment proximal to inflammation. Both the inflammatory process and MS may induce proinflammatory molecules (i.e., COX-2), cytokines and chemokines (i.e., IL-6, IL-8, osteopontin), and pain mediators (i.e., prostaglandin E2, nerve growth factor). CD is also associated with microbiota dysbiosis in the lumen. The altered gene expression and dysbiosis are responsible for inflammation, immune responses, barrier dysfunction, fibrosis, visceral pain, and dysmotility in CD. EEN treatment with liquid diet is found to reduce inflammation and immune responses, improve barrier function and motility, and restore microbiota composition and diversity. Recent studies found that these benefits may be due to EEN’s effect to attenuate MS and MS-dependent pro-inflammatory gene expression. Notes: EEN, exclusive enteral nutrition; CD, Crohn’s disease.

Transmural inflammation in CD is associated with inflammatory cell infiltration, edema, fibrosis, stenosis, and distention, which present increased mechanical stress (MS) to the bowel wall [45]. Recent studies discovered that MS can be both a consequence and a cause of transmural inflammation and fibrosis in CD [131,132,133]. In a murine model of CD, we found that MS initially results from inflammation and stenosis, but later interacts with inflammation to further disease progression [45]. The gut lumen normally experiences transient MS due to bowel movements during the passage of solid food. Our previous research in a rat obstruction model showed that MS alters gene expression in the distended colon segment, with a significant increase of pro-inflammatory mediators (130). A similar increase in pro-inflammatory molecules is also observed in the distended segment proximal to inflammation in the CD model. We found that EEN with a liquid diet reduced intestinal inflammation and decreased the expression of mechano-sensitive pro-inflammatory genes, i.e., IL-6 and COX-2 [45,132]. While the significance of Th17 immune responses in CD progression is well-established, the specific cellular processes driving their development and persistence are not fully understood. Our research in the CD model has shown a strong connection between Th17 immunity and the distended proximal segment of the colon. Interestingly, EEN dramatically reduced mechanical distention and significantly decreased the Th17 response in the proximal segment. Consistent with this observation, our rat model of mechanical obstruction, which increases MS, displayed an elevated Th17 population in the mechanically distended colon, suggesting a clear link between MS and Th17 responses. Additionally, we observed upregulation of mechano-sensitive genes known to promote Th17 immunity (i.e., IL-6 and osteopontin) in the proximal segment. EEN treatment abolished the increase of mechano-sensitive genes. Importantly, neutralization of IL-6 blocked Th17 response in the CD model [45]. These findings suggest that EEN may reduce inflammation and immune response by eliminating MS and MS-induced pro-inflammatory mediators (Figure 1, Table 1).

Increased MS has been found to contribute to dysmotility, visceral pain, and microbiota dysbiosis in conditions with bowel stenosis and distention [134,135], which are commonly encountered in CD. Bowel distention also leads to barrier dysfunction [135]. Johnson et al. found that EEN treatment not only prevented mechanical distention in CD-like colitis rats, but significantly improved colonic motor function [132]. They found that this beneficial action may be due to EEN effect to attenuate mechano-sensitive expression of COX-2 in colonic smooth muscle cells, as MS led to marked expression of COX-2 in the smooth muscle cells and COX-2 inhibition partially restored colonic motor function in the distended colon [132]. As EEN treatment eliminates MS, the documented benefits of EEN on disease activity, barrier function, and microbial health in CD patients may also be attributed to the elimination of MS by EEN (Figure 1). Further studies are warranted to better understand the regulatory mechanisms of MS in Th17 immune responses, barrier function, and neuromuscular activities in the GI tract. Nevertheless, these findings will shed light on how EEN works in CD.

## 5. Conclusions and Future Directions

Among all the tested nutritional therapies, EEN has been well-documented to have anti-inflammatory and healing effects in CD through various potential pathways, including altering gut microbiota and their metabolites, restoring the gut barrier, direct anti-inflammatory action, and indirect anti-inflammatory action by eliminating mechanical stress. It is possible that these mechanisms involve a complex interaction between the body’s pathophysiological and immune responses in the gut and the environment within the intestines. However, recent studies found that these benefits may be due to EEN’s action to attenuate mechanical stress and mechanical stress-dependent pro-inflammatory gene expression.

It will be very important to further understand the cellular and molecular mechanisms involved in mechanical stress-induced pro-inflammatory gene expression in the gut. It is equally important to identify other mechano-sensitive genes in the gut and determine their biological functions in health and diseases. The outcomes will help us to identify new therapeutic targets for CD and other conditions with increased mechanical stress in the bowel wall.

The clinical efficacy of other nutritional therapies in CD is not well-established, and mechanisms for the potential benefits are unclear. More rigorous clinical trials are needed to determine the efficacy of the existing and novel nutritional therapies. Further basic and translational studies are warranted to understand the mechanisms of action of these treatments.

Gut microbiota plays a critical role in human intestinal homeostasis, and dysbiosis is associated with development and progression of CD. As microbiota is heavily involved in the metabolism of nutrients and nutritional therapies, further studies are warranted to determine if and how microbiota is involved in the effects of nutrients and nutritional therapies in CD.

A better understanding of the mechanisms of action of nutritional therapies will help to improve patient outcomes, reduce dependence on medications, tailor treatment options precisely and personally, and identify factors predicting responsiveness to medications and nutritional therapies in CD.

## Figures and Tables

**Table 1:** Different nutritional therapies and their effects on inflammation and microbiota in CD.

Diet Type	Microbiome Changes	Pathological and Clinical Impacts
SCD	↑ Microbial diversity, ↑ Clostridia species [63] ↑ Blautia, a Lachnospiraceae [80] species, *Faecalibacterium prausnitzii*, *Roseburia hominis*, *Roseburia intestinalis*, *Anaerobutyricum hallii*, and *Eubacterium eligens* ↓ *Escherichia coli*, *Faecalibacterium prausnitzii* [81]	↓ Inflammation ↑ Mucosal healing [60,61,62]
FODMAP	↓ Bifidobacterium species ↓ *Faecalibacterium prausnitzii* [70,82,83]	↑ Improved gastrointestinal symptoms [66,68]. ↓ Inflammation [84]
CDED	↑ Microbial diversity ↓ Proteobacteria ↑ Firmicutes [53,54,55,56,57]	↑ Mucosal healing ↑ Barrier integrity ↓ Inflammation [75], [85,86]
EEN	↑ [44] or ↓ [87,88,89] Microbiome diversity ↓ F. Prausnitzii, Bacteroides/Prevotella group species, Proteobacteria, *Dorea longicatena*, *Blautia obeum,* and *Bifdobacterium longum* [87,88,90,91,92,93,94] ↑ Firmicutes, Clostridium V [75] ↑ *Lactobacillus* and *Dubosiella* ↓ Bacteroides and *Enterorhabdus* [44]	↑ Barrier integrity [80,95,96] ↓ Inflammation [37,38,39,40,41,42,43] ↓ Th17 immune response [45], ↓ Short-chain fatty acids [97] ↓ Mechanical stress [45,79]

Notes: ↑: Increased; ↓: Decreased.
